# Progressive Methods in Studying the Charred Layer Parameters Change in Relation to Wood Moisture Content

**DOI:** 10.3390/polym14224997

**Published:** 2022-11-18

**Authors:** Dominik Špilák, Andrea Majlingová

**Affiliations:** Department of Fire Protection, Faculty of Wood Sciences and Technology, Technical University in Zvolen, T. G. Masaryka 24, 960 01 Zvolen, Slovakia

**Keywords:** fire resistance, charred layer, finite element model, ANSYS

## Abstract

The aim was to investigate the relationship of charred layer parameters (also wood fire resistance) and moisture content of European larch (*Larix decidua* L.) wood. For this purpose, finite element model (FEM) was developed. To develop FEM, ANSYS software and transient thermal analysis were applied. To validate developed FEM, the medium-scale fire tests were provided in the laboratory chamber. In the fire tests the beams made of larch wood have undergone the thermal loading with radiant panel. The FEM validation results showed very strong correspondence of numerical and experimental results, when achieving the overall accuracy of 93.4%. Validated FEM was further used to determine the relationship between the larch beams moisture content and formation of charred layer, i.e., its parameters. The results from the simulation pointed out the fact, the wetter the wood, the higher its fire resistance. This is very important information for studying the formation of a charred layer and a layer of degraded wood. After increasing the moisture content from 10% to 30%, the area of the charred layer decreased by approximately 20%. The area of degraded wood decreased by almost 30%, so it can be stated that the area of the charred layer of wood and degraded wood decreases exponentially with increasing wood moisture content.

## 1. Introduction

Slovakia is a country with an increasing and relatively high share of land covered by forests, which allows for the significant increase of the contribution of wood as a construction material to the production and development of building structures. Wood is well known, mostly for its structural and mechanical properties and suitability for constructions [1]. For wooden building structures, this material meets the increasing demand of present and probably the foreseeable future, because of its environmental friendliness. It is suitable material to be used in housing, industrial production, and constructions.

Wood is a complex heterogeneous colloidal system of substances. It consists of main and accompanying components. Among the main three components belong hemicelluloses, cellulose, lignin. The behavior of the wooden structure in fire is influenced mostly by heat transfer, heat conduction and method of thermal loading. The strongly thermally loaded wood undergoes thermal degradation [1]. Thermal loading on the surface and conducted heat inside the wood causes chemical and physical changes in it. There is a difference in resistance to fire of the elementary wooden building blocks. The hemicelluloses are the least resistant to thermal loading and lignin is the most resistant [2].

The thermal degradation process of wood is characterized by pyrolysis processes which results in forming the charred layer. The typical wood pyrolysis manifestations are mass loss, wood coloration to brown, and formation of combustion by-products (gases) [3]. Pyrolysis is a complex process depending on, e.g., oxygen concentration, wood moisture content, and position of the sample (wooden beam) to the thermal loading source [4]. Wood moisture content significantly impacts on the results of fire test. It is reflected by temperatures and the flame spread index mostly. Wetter wood needs more energy for reaching the flash point or autoignition temperature, i.e., the flame spread index is lower.

Pinto et al. [5] stated that the insufficiency of oxygen in wood combustion process results in the wood decomposition into several components as well as charred layer formation.

When performing the fire tests, test samples with required moisture content must be prepared. For any type of fire test, there are predefined moisture contents of the samples to be tested. However, also other parameters must be considered, e.g., product installation site, average atmospheric condition (temperature, relative air humidity) at this site.

**Figure 1:**
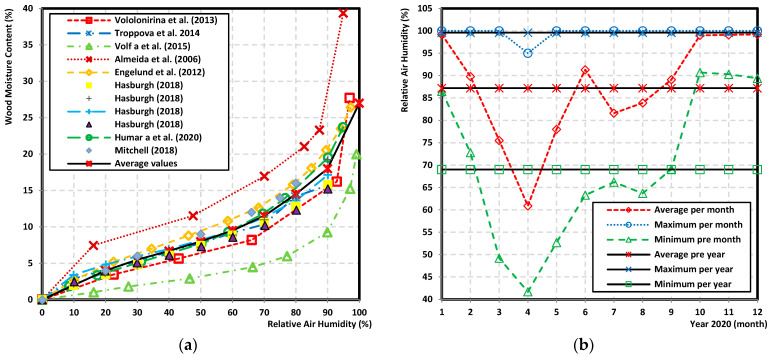
Wood moisture content and relative air humidity. (**a**) Wood moisture content in dependence on relative air humidity. (**b**) Relative air humidity in Slovakia in 2020.

The relationship between the atmospheric conditions, i.e., relative air humidity and the moisture content of wood observed at a specific temperature is called the sorption isotherm. It represents the wood moisture content limiting (critical) values [6,7,8,9,10]. As stated by several authors, e.g., [11,12], higher the relative air humidity, higher the wood moisture content (Figure 1a).

Using the average relative air humidity values, predictions of moisture content of the wood used in the building structures can be developed at specific reliability level. Knowledge about the wood moisture content is necessary for achieving accurate results of wood heat transfer and conduction calculations.

The relative air humidity (%) is the ratio of the amount of humidity to the maximum possible quantity at the same temperature. In the atmosphere, water is found in all three states. There is a constant exchange of humidity between the active surface and the atmosphere by means of turbulent flow and molecular diffusion. Fluctuations in relative air humidity are generally related to periodic changes in air temperature. The trend of the average relative air humidity values in 2020 is shown in Figure 1b. Data provided by the Slovak Hydrometeorological Institute showed that the relative air humidity from 42 to 100% in Slovakia in 2020. These values correspond with the wood moisture content values which were in the range of 8 (9)% to 25%. 

Char is well and easily identifiable part of the wood exposed to fire which is formed during the thermal loading at specific temperatures. It is often described as a black porous solid part of the wood. Its composition is formed mostly by elemental carbon [5]. Many authors stated that it consists of several layers. For example, Fonseca and Barreira [13] stated that a wooden cross-section which undergoes the thermal loading is formed by the following zones: charred layer, charcoal boundary, pyrolysis layer, pyrolysis boundary, and intact wood. Charred wood is bounded by the transition between those zones. This transition is usually located at the isotherm of 300 °C; it is called the char-line. When measuring the depth of the charred layer, the distance between the outer edge of the sample surface and the position of the char-line must be considered. 

In wood pyrolysis, two thermal phenomena were found [14], i.e., charring at low temperatures and at high temperatures. Thermal loading with low-temperatures results in degradation of hemicelluloses. There is the carbonaceous residuum (char) formed in temperature range 95 to 120 °C [15]. In this temperature range, the cellulose passes the glass transition phase and softens [16]. Lignin degradation occurs at temperature range of 55 to 170 °C [17]. At temperatures above 200 °C, there is the discoloration and primary and secondary pyrolytic reactions are found. The temperature ranges of 120–180 °C, 200–260 °C, 200–260 °C, and 220–315 °C [3,18] are the operating (high) temperatures at which the degradation of hemicellulose and charring process occur. This process is influenced by the properties of material and thermal loading [14]. The cellulose thermal degradation occurs in the following temperature ranges: 240–350 °C, 250–350 °C, 315–400 °C, 280–400 °C and 300–350 °C [3,19]. The pyrolysis the lignin was observed in the temperature ranges of 110–400 °C [19], 280–500 °C [20], and 225–450 °C [21].

The charred layer of wood shows weak thermal conductivity, i.e., the process of heat transfer is slower when reaching the other layers of wood [22]. Blass [23] or Harper [24], stated, the charred layer thermal conductivity values are lower than the intact wood thermal conductivity. Su et al. [25] stated that, the deeper the charred layer, the better the thermal insulation parameters of wood and lower the charring rate values of the remaining wood cross-section at the same time. In the EN 1995-1-2 (Eurocode 5) [26], the rate of charring is assigned to wood and wood-based building materials products according to the type. The wood rate of charring is also influenced by the wood moisture content, orientation of surface, movement of air, thermal loading, cracks existence, and other [27]. 

When studying the behavior of structures exposed to thermal loading, i.e., fire, the heat transfer analysis (HTA) is often applied. In HTA, also modelling tools are deployed, mostly for prediction the behavior of pyrolysis front. The pyrolysis front is a transition zone which separates the fire undamaged (intact) wood layer and the charred layer. Previously, several calculation methods, both, simple and advanced, were applied and published. Pyrolysis can be characterized as a nonlinear process, showing signs of inherence and complexity. However, when modelling the pyrolysis process, there are used greatly simplified and linear models, e.g., as specified in Eurocode 5. This one is a single parameter model based on the values of nominal charring rate. The nominal charring rate (*β0)* describes the charring rate at 60 min of standard thermal loading. The nominal charring rate is affected by the wood species (softwood or hardwood), its character (solid-sawn wood or structural element), its moisture content, and the direction of thermal loading (i.e., parallel to grain or perpendicular). The nominal charring rates related to various wood products are provided by several sources, also in Eurocode 5. Using the information on nominal charring rate, we can quantify the charred layer depth, as a function of time.

The depth of charred layer is an important parameter for setting the specific wood species fire resistance. Fire resistance is a key parameter in designing the building’s fire safety measures to ensure a sufficient time frame for the safe evacuation of all occupants. In fire investigation, the depth of charred layer can be used as an evidence of fire spread. Having the information on relative values of charred layer depth and area, the identification of longest thermally loaded materials or construction parts in enclosure space is possible. Knowledge of the relative depth of the charred layer is key for locating areas where damage was more severe due to the position of the heating source, enclosure ventilation conditions, or the location of the fuel in the enclosure.

When providing the fire tests, especially tests of fire resistance of structures, the worst conditions that may occur at the product installation site must always be considered. Thus, the values at which the wood moisture content is the lowest are represented by the values of lowest activation energy, which are required to ignite the wood. This results in a shorter time of wooden structure resistance to fire, i.e., thermal loading [9]. Based on the previous information, the wood moisture content is important test variables that affect the formation of charred layer of wooden construction elements. The aim of the study is to investigate the relationship of charred layer parameters (also wood fire resistance) and moisture content of European larch (*Larix decidua* L.) wood.

## 2. Materials and Methods

The methods used follows the study’s main objective, which is to investigate the relationship between the moisture content of wood and charred layer parameters of wooden beams thermally loaded with radiant panel. The finite element analysis (FEA) was applied to study it. First, the medium-scale fire tests with larch wooden beams were provided, followed by development of a finite element model (FEM) with material properties and geometry corresponding to medium-scale fire tests. Afterwards, there was a validation of the FEM model provided with a real fire test. The impact of wood moisture content on the charred layer parameters was studied when changing the material properties in FEA. 

### 2.1. Medium-Scale Fire Tests of Wooden Beams

To validate the developed FEM, there were implemented the medium-scale fire tests with wooden beams. The thermal loading with a duration of 60 min for each sample was applied. The wooden beams were made of European larch (*Larix decidua* L.) harvested in the Technical University in Zvolen Forest Enterprise territory, Central Slovakia during the winter 2020. The dimensions of the wooden beam’s samples were as follows: length of 1000 mm along with a height and width of 190 mm ± 5 mm, because of the square cross-section of the samples. The length of the sample part which was directly exposed to the source of thermal loading was of c.a. 500 mm. A total of 4 test samples were subjected to medium-scale fire tests which complied the Eurocode 5 requirements for testing the fire resistance of wood and wooden constructions. The first fire test was provided only to set up the test procedure and test equipment used.

**Figure 2:**
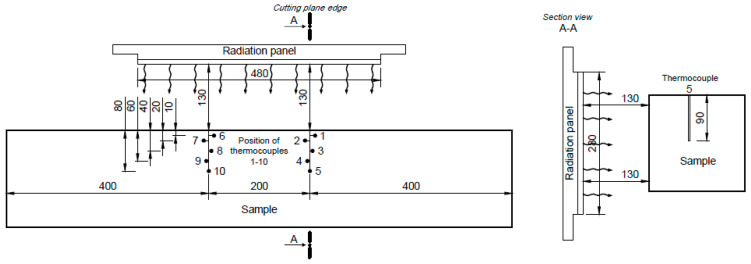
The experimental setup showing the position of thermocouples used.

Providing the medium-scale tests was a key requirement to acquire the temperature courses and the charred area parameters for FEM development. As already mentioned, the duration of the medium-scale fire tests was set to 60 min. There were predetermined the positions, where the temperature series were recorded during the thermal loading. The temperatures were recorded by the thermocouples of NiCr-Ni type (Omega Engineering Inc., Norwalk, CT, USA) which allows for the recording of the temperature in the range −40 °C to +1200 °C. In Figure 2, the position of the thermocouples is introduced as they were positioned in the test sample.

A total of 10 thermocouples were placed in two groups of 5 thermocouples at a depth of 90 mm for each sample. The positions of thermocouples were similar in both groups. The thermocouple T1 was of 10 mm far from the edging of the thermally loaded side of the sample, the thermocouple T2 of 20 mm, the T3 thermocouple of 40 mm, the thermocouple T4 of 60 mm and the thermocouple T5 of 80 mm. The thermocouples were inserted into a pre-drilled holes with a diameter of 3 mm and a depth of 90 mm. To ensure drilling accuracy and reduce deviations, a drilling template was used. Groups of thermocouples were 200 mm apart. For recording and processing, the temperature values measured by the thermocouples the AHLBORN ALMEMO 2290-8710 V7 datalogger (Ahlborn Mess- und Regelungstechnik GmbH, Holzkirchen, Germany) was used. 

**Figure 3:**
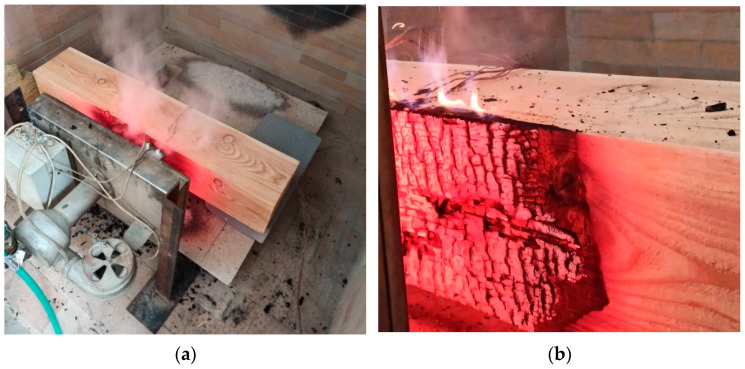
Medium-scale fire tests: (**a**) test equipment and (**b**) laminar flames.

The size of the radiation panel heating area was 480 × 280 mm^2^, the emitted energy was produced by the burning propane–butane gas with a constant flow of 15 m^3^∙h^−1^. The test equipment used is shown in Figure 3a. The samples were placed at 130 mm far from the radiation panel. The distance of 130 mm represents the minimum distance of the sample from the radiation panel, when expected that the laminar flames will appear (Figure 3b). At the end of each test, all the samples were cooled. Slices with a width of 10 mm were cut from the centre of the test samples to determine the charred layer area.

**Figure 4:**
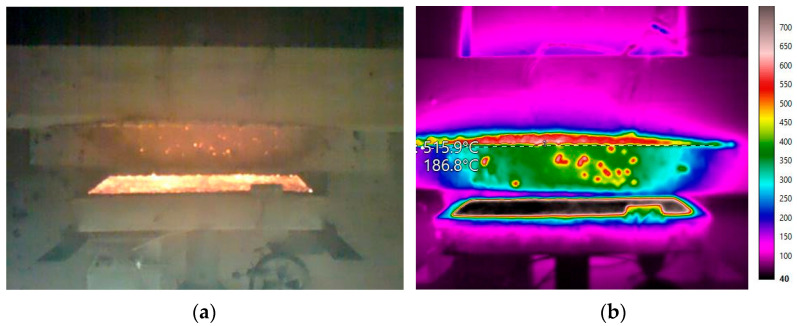
The outputs from optical (**a**) and thermal (**b**) imaging camera.

A Fluke RSE600 thermal (infrared) imaging camera (Fluke Corporation, Everett, WA, USA) with temperature measurement range −10 to +1200 °C was used to measure the temperature on the entire visible surface of the sample and on the radiation panel (Figure 4). The thermal imaging camera was placed on a tripod above the sample at height of 2 m. The temperature courses recorded by the thermal imaging camera (values for each pixel of the image inn interval of 0.1 s) were used to detect the temperature of the radiation panel to be entered in simulation as well as for the FEM validation. 

### 2.2. Selection of Fire Models

According to Frangi, Erchinger and Fontana [28], Zhang et al. [29], Molina et al. [30], Couto et al. [31], Regueira and Guaita [32], and Dúbravská et al. [33], complex reactions involved in wood burning were simplified for fire model development purposes. The fire models use the transient heat conduction in the wood material for this purpose. The authors used thermal analysis to investigate the behavior of various wooden construction element types during thermal loading. For this purpose, they used system ANSYS. 

In this study, we assumed that having information on temperature course could help to determine the depth and area of the charred layer and the intact wood of the sample resistant to thermal loading for at least of 60 min. 

ANSYS 2022/R2 (ANSYS, Inc., Canonsburg, PA, USA) and transient thermal analysis were used in this study to develop the FEM for larch wooden beams. 

The Fourier’s law is used in ANSYS (ANSYS, Inc., Canonsburg, PA, USA) for governing the relationship between heat flux and temperature [34]:

(1) q=−k·∇T

where, 𝑞 is the heat flux, ∇𝑇 is the temperature gradient and 𝑘 is the thermal conductivity, which represents the ability of material to transfer heat by conduction.

Thermal conductivity can also be defined as the heat flux transmitted through a material due to a unit temperature gradient under steady state conditions. In the thermal analysis, the steady state is considered, not the effect of time. It is a material property which is independent on the geometry of the object of conduction. 

The thermal conductivity of wood materials changes with temperature. For materials where thermal conductivity changes with temperature, Fourier’s law is modified as follows, where the thermal conductivity *k* is a function of temperature *T* [35]:

(2) q=−k(T)·∇T

Equation (2) describes steady-state heat conduction in a one-dimensional space. For materials with different thermal conductivities in different directions, Fourier’s equation is written as:

(3) $$\left[\begin{array}{c} q_{x}\\ q_{y}\\ q_{z} \end{array}\right]=\left[\begin{array}{ccc} k_{xx} & k_{xy} & k_{xz}\\ k_{yx} & k_{yy} & k_{yz}\\ k_{zx} & k_{zy} & k_{zz} \end{array}\right]\cdot \left[\begin{array}{c} \frac{dT}{dx}\\ \frac{dT}{dy}\\ \frac{dT}{dz} \end{array}\right]$$

Heat flux q is a vector. It can have various values in different directions. Thermal conductivity k is a tensor, and ∇T is a vector.

The dependence of thermal conductivity on the temperature is not always simple, considering a more complex 2D or 3D geometry, therefore, for these cases the Newton–Raphson method [35] is applied. The equations do not include time for solving heat transfer problems. 

The difference between steady state of heat transfer and transient state of heat transfer is in the fact, that the most applied thermal loadings in the transient thermal analysis are functions of time (density, specific heat, and temperature). The main equation for transient heat conduction through a solid is specified in the following Equation (4) including the thermal equilibrium [35,36]:

(4) $$k\times \left(\frac{\partial^{2}T}{\partial x^{2}}+\frac{\partial^{2}T}{\partial y^{2}}+\frac{\partial^{2}T}{\partial z^{2}}\right)+q=\rho \times c\frac{\partial T}{\partial t}$$

(5) $$k\nabla^{2}\times T+q=\;\rho \times c\frac{\partial T}{\partial t}$$

where, ρ is density of a material, c is specific heat capacity, *k* is thermal conductivity, $\bar{q}$ is heat flux and T is thermodynamic temperature. In the equation for transient heat conduction, the expression on the right side of the equation ($\rho \times c\frac{\partial T}{\partial t}$) represents the rate of energy storage in the body.

### 2.3. Modeling the Behavior of Structural Elements in Fire in ANSYS

**Figure 5:**
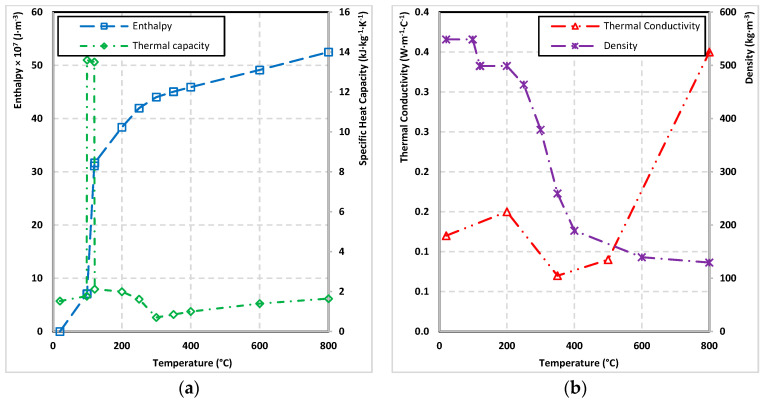
The properties of European larch: (**a**) enthalpy and specific heat capacity, and (**b**) thermal conductivity and density.

The properties (density, thermal conductivity, enthalpy–enthalpy approach) of European larch wood was entered in the initial phase of model design. The initial density of European larch samples at 20 °C with 0% moisture content were determined to be 489.9 kg·m^−3^ by gravimetric method. The small samples for determining the density were made from different parts of the unused medium-scale sample (the dimensions of the small samples were 5 × 5 × 2 cm^3^). The moisture content of the wood was determined using the difference in the weight of the small samples before and after drying to 0% moisture content. The moisture content was set at 10% with a deviation of 0.1%. According to Eurocode 5 [26] graphs of wood density and thermal conductivity dependency on temperature were created (Figure 5b).

In Ansys, the definition of enthalpy is adjusted to represent the energy absorbed by the “unit volume” of the body. For wet wood, the derivation of the enthalpy curve is based on two different effects of heat, heating the wood, and evaporating the free water contained in the wood. After reaching a temperature of 100 °C, the contained water is converted into water vapor. This phase change consumes most of the heat received, which results in a slowdown in the heating of the wood.

The Equations (6)–(8) [37] were used to calculate the wood enthalpy values (Figure 5a). In the calculation of wood enthalpy, the values of wood specific heat capacity (according to Eurocode 5 [26]) were involved, see Figure 5a.

(6) $$H_{T_{1}\;}=H_{T_{0}}+c_{dw}\times \rho_{dw}\times (T_{1}-T_{0})+\frac{w}{100}\times c_{w}\times \rho_{w}\times (T_{1}-T_{0});\;0<T_{1}<99$$

(7) $$H_{T_{2}}=H_{T_{1}}+c_{dw}\times \rho_{dw}\times (T_{2}-T_{1})+\frac{w}{100}\times H_{evap}\times \rho_{w};\;99\leq T_{2}\leq 120$$

(8) $$H_{T_{3}}=H_{T_{2}}+c_{sd}\times \rho_{sd}\times \left(T_{3}-T_{2}\right);120<T_{3}<1200$$

The starting point of the volume enthalpy curve $H_{T_{0}}$ is equal to 0. Furthermore, according to the above-mentioned equations, $c_{dw}$ is thermal capacity of dry wood, $c_{w}$ is the thermal capacity of water, $\rho_{dw}$ is density of the dry wood, $\rho_{w}$ is density of water and w is the moisture content of the wet wood. When involving $H_{T_{0}}$ in simulations, computer programs usually do not tolerate zero numbers. In those cases, the value $\underset{n\to \infty }{\lim}\frac{1}{n}$ must be entered. 

Equation (6) is used to calculate the dry wood enthalpy and the wood moisture content at temperature *T1*. 

Equation (7) is a calculation of enthalpy, when the state of water changes up to the temperature *T2*. In this equation, $H_{evap}$ is enthalpy of evaporation. 

Equation (8) allows for the calculation of the enthalpy of dry wood at temperature *T3*. 

Enthalpy calculations provided, were carried out separately, considering any change in wood properties.

#### 2.3.1. Geometry of the Model, Meshing and Simulation Settings

The FEM was created in the “SpaceClaim” environment. The model was fully in correspondence with the elements (samples) used in the medium-scale tests. To simplify the model as much as possible, the final FEM does not consider the entire medium-scale fire tests settings (supporting structure, environmental condition). However, their absence did not affect the simulation results and accelerated the calculations on the other hand.

Since wood is a heterogeneous material, its thermal conductivity is different in the direction of the fibers and perpendicular to the wood fibers. Since the wooden beam is heated evenly to almost one side of it and heat transfer perpendicular to the fibers is dominant, the difference in results when using isotropic thermal conductivity or orthotropic thermal conductivity is negligible. Using isotropic thermal conductivity simplifies and speeds up the calculations. Applying the “Patch Conforming Method” created a different mesh for wooden beams and radiation panel. The tetrahedral mesh with element size 5 mm was created for wooden beams, while the hexahedral mesh with element size 1 mm was created for the radiation panel. The total number of elements and nodes for wooden beams was of 550,000, i.e., 3,600,000, respectively. The connection region in the model was created manually, representing the thermal connection between the radiation panel and the thermally loaded surface of the wooden beam.

The initial temperature was set to 24 °C based on the results of the fire tests provided. The simulation duration was set to 3600 s. In addition, the numbers of sub-steps and iterations were set based on the real fire tests data. The duration of a sub-step was of 10 s. The maximum number of iterations was set to 1000. Since the convergence criterion was not met in the initial phase of the simulation, the value of thermal convergence was changed from 1.5% to 10%. Although, ANSYS evaluated it to be an error, due to the imbalance in the system. This was caused by a sharp temperature rise in one sub-step, which ANSYS could calculate only with a certain deviation, exceeding the pre-set criterion. 

As a boundary condition of the simulation in ANSYS, uniform surface radiation panel with temperature of 955 °C was set. The radiation panel surface temperature values were recorded by the thermal imaging camera when providing the real fire tests. The surface temperature of the radiation panel was approximately constant during the entire fire test (±10 °C).

#### 2.3.2. Method of Solution and Processing of Results

Using the “Temperature” function, the temperatures were recorded in predefined positions as used in the fire tests provided. Furthermore, they were processed into the tabular form. The outputs of the simulation also included images of sections of each sample, which were further used to determine the depth and area of the charred layer. According to Eurocode 5 [26], Zhang et al. [38], Couto et al. [31], Regueira and Guaita [32], and Dúbravská et al. [34], reaching the temperature of 300 °C was considered the point for determining the depth of charred layer.

#### 2.3.3. Validation of Applied Fire Models Using the Results of Medium-Scale Fire Tests

The validation of an FEM is a process of verification of the exactitude of the models and the material characterization associated with the existing experimental results [39]. The validation of the developed FEM was provided based on the temperature data, which were measured by the thermocouples and recorded by the thermal imaging camera and charred layer physical models from samples that undergone obtained the thermal loading from in the medium-scale fire tests. The starting point for FEM validation was a comparison of results from real fire tests and simulations. The temperature profiles recorded in different depths and area of the charred layer were compared when validation the FEM. The key task was to achieve the highest possible accuracy of the simulation.

#### 2.3.4. The Effect of Moisture Content of Wood on the Formation of a Charred Layer of Wood

**Figure 6:**
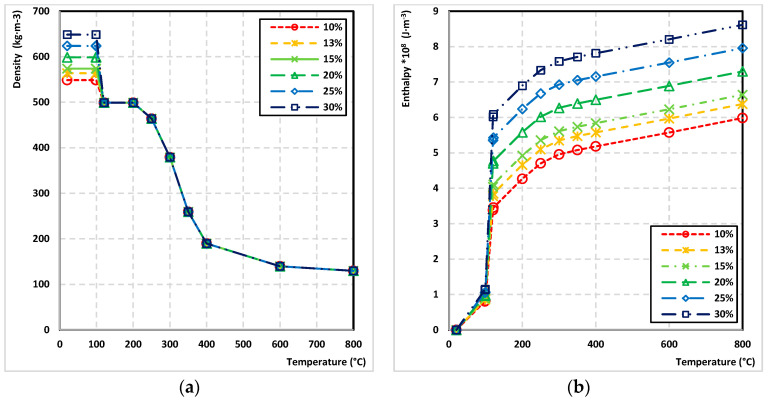
The properties for different moisture content in wood: (**a**) density and (**b**) enthalpy.

For investigating the effect of moisture content of wood on the wood charred layer parameters, the ANSYS software and validated FEM were used. A total of 6 transient thermal analysis of heat transfer in wood thermally loaded were provided. They were used to determine the area of the charred layer (temperature reached minimum 300 °C during the fire test) and the area of wood degradation, i.e., pyrolysis zone, (temperature was in the range of 100–300 °C during the fire test). In each analysis, the moisture content of the wood varied (10, 13, 15, 20, 25, 30%). The wood moisture content parameter was implemented in the ANSYS simulation by changing the wood density and enthalpy corresponding with applied wood moisture content (Figure 6). The initial density of the wood at the specified moisture content was as follows: 10%–548.8 kg·m^−3^, 13%–563.8 kg·m^−3^, 15%–573.7 kg·m^−3^, 20%–598.7 kg·m^−3^, 25%–623.6 kg·m^−3^, and 30%–648.6 kg·m^−3^.

## 3. Results

### 3.1. Result of the Validation

**Figure 7:**
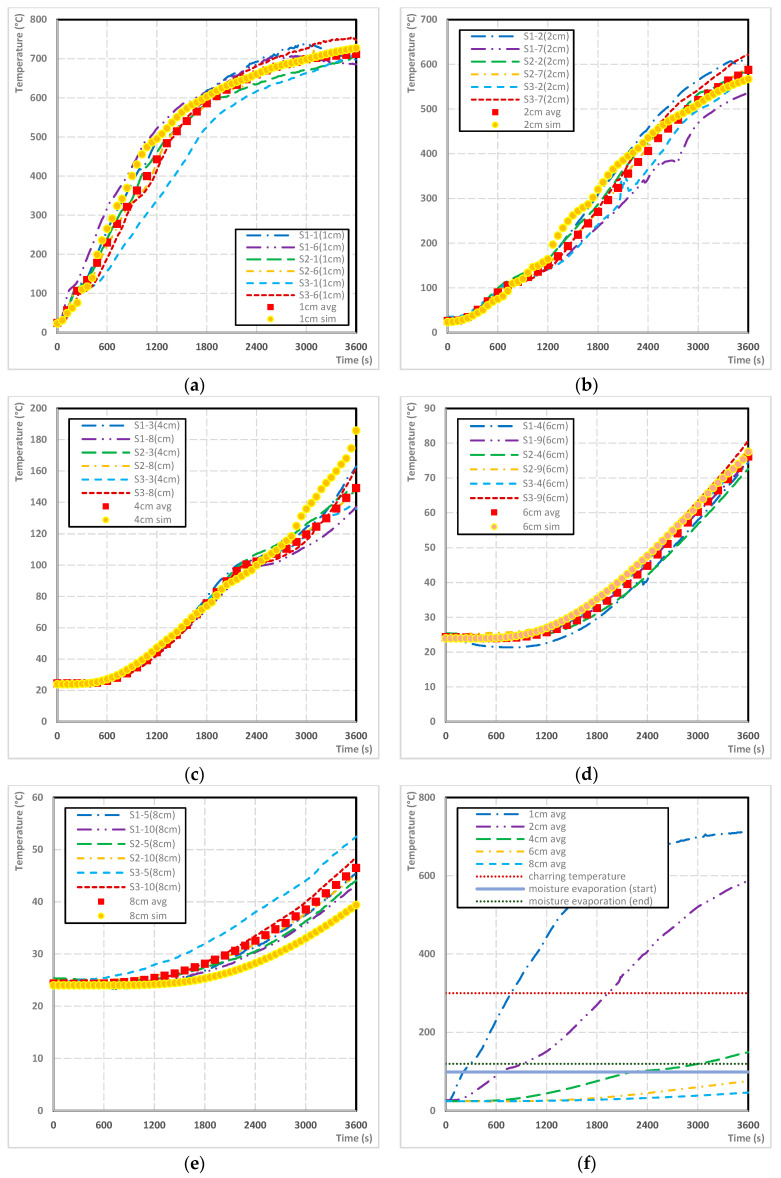
A comparison of temperature profiles—real test and simulation: (**a**) 1 cm depth, (**b**) 2 cm depth, (**c**) 4 cm depth, (**d**) 6 cm depth, (**e**) 8 cm depth, and (**f**) comparison of average temperature profiles.

To demonstrate the results related to the comparison of medium-scale fire tests findings and simulations outputs, there were created the charts. There are further introduced the charts showing the temperature profiles and images of individual sections showing the depth of charred layer and graphs introducing the charring rate. Each chart contains a record of the temperature got from real medium-scale fire tests of wood beams with 10% moisture content in a specific position and depth, the average value and the temperature profile obtained from simulations (Figure 7). 

Based on the comparison of the average temperature from the medium-scale fire tests and the simulation the accuracy of prepared FEM is 93.4% with an average deviation of 1.7%. 

**Figure 8:**
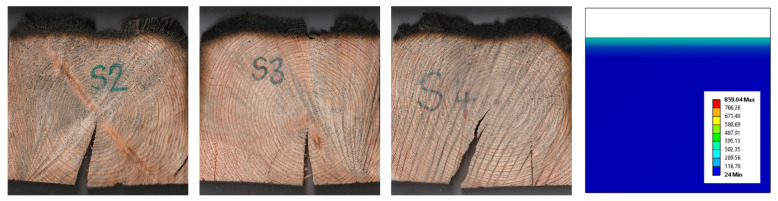
Cross section of samples: comparison of results from medium scale fire tests and simulation.

After the completion of medium-scale fire tests and making cross-sections, the individual sections were scanned into a computer, where the area of the charred layer was measured using CAD software. The boundary of the charred layer is clearly visible after increasing the color contrast, while at several approximations (10≤) the interface was sharp enough. The charred layer from simulation was measured using ANSYS software as the area of the sample where the temperature was higher than 300 °C. The average charred area was measured at 5896 mm^2^. The area determined by simulation was 5846 mm^2^. FEM determined the area of the charred layer with an accuracy of 99.1% (Figure 8).

Based on the comparison of data from medium-scale fire tests and simulation, we can consider the FEM as validated and applicable for further research. 

### 3.2. Effect of Moisture Content

Based on the validated FEM, the influence of different wood moisture content on the formation of a charred layer and the area of degraded wood was investigated. The influence of moisture content on charred layer formation during the thermal loading was included into the FEM by changing the material (wood) properties (enthalpy and density) associated with the specified moisture content. 

The results of the modeling and simulation of thermal loading of larch wooden beams with dimensions as specified in Section 2.1 and different densities are as follows. For wooden beams with a moisture content of 10%, the area of the charred layer was of 5846 mm^2^ and the area of degraded wood was 3172 mm^2^. At a moisture content of 13%, the area of the charred layer was 5629 mm^2^ and the area of degraded wood was 2997 mm^2^. At a moisture content of 15%, the area of the charred layer was of 5472 mm^2^ and the area of degraded wood was of 2935 mm^2^. At a moisture content of 20%, the area of the charred layer was of 5329 mm^2^ and the area of degraded wood was 2789 mm^2^. At a moisture content of 25%, the area of the charred layer was 5094 mm^2^ and the area of degraded wood was of 2670 mm^2^. At a moisture content of 30%, the area of the charred layer was 4715 mm^2^ and the area of degraded wood was of 2295 mm^2^. 

**Figure 9:**
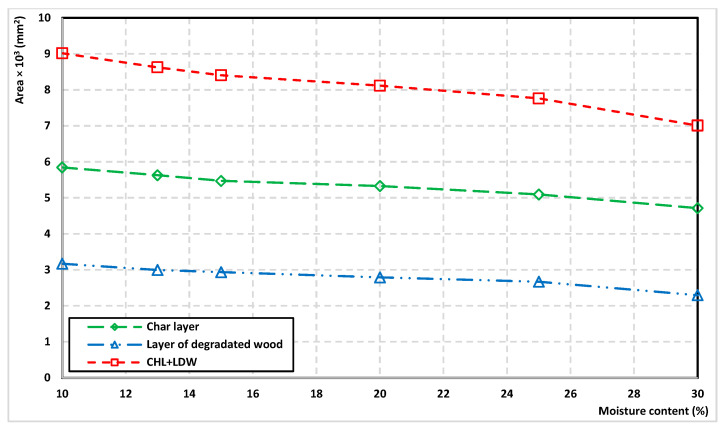
The degradation of wood vs. moisture content.

It is clear from the results that the area of the charred layer of wood and degraded wood decreases exponentially with increasing moisture content (Figure 9). The shape of the charred layer and the layer of degraded wood are the same even when changing moisture content. 

The results show that with an increase in wood moisture content from 10% to 30%, the area of the charred layer decreased by 19.34%. The area of degraded wood decreased by 27.64%.

## 4. Discussion

The results show a significant influence of moisture content of wood on the charred layer and layer of degraded wood parameters. It is logical that with increasing of moisture content of wood, the wood density increases. The increasing density is associated with the amount of energy required by the material to raise the temperature by 1 °C. Based on the previous, the density has the effect on the temperature profiles of wooden beams exposed to fire.

The charred layer has a significant impact on determining the fire resistance of wooden structural elements. However, the layer of degraded wood has also a significant impact since wood significantly loses its strength properties when undergoing thermal loading, which is also stated in Eurocode 5 and confirmed by the studies of several authors. Schaffer [39], Glos and Henrici [40], Konig and Walleij [41], Konig [42], Zeeland [43], Gerhards [44] and Tabaddor [45] report that the strength of wood in pressure decreases due to the increase in temperature of wood, significantly already in temperatures range 20 °C to 300 °C, starting with the maximum values to almost zero. Similarly, the modulus of elasticity of wood decreases significantly with increasing temperature, as shown by several authors (Eurocode 5 [26], Jong and Clancy [46], Janssens [47], Schaffer [48], White et al. [49], and Ostman [50]). 

According to the results of simulations, the wood area losing the strength properties decreased by 22.26%. Significant decrease has an impact on the fire resistance of wooden construction elements. By increasing the wood moisture content, the fire resistance increases. 

Fire resistance tests of wooden structural elements must be carried out at a precisely defined moisture content of the samples (Eurocode 5). In the presented study and its results, it was demonstrated that the materials (wood) have a much higher moisture content values at the installation sites as specified for fire tests implementation in the Eurocode 5 and other standards. This could be a problem, because when conducting fire tests to determine the fire resistance of the materials, the fire tests are carried out in accordance with predefined conditions (moisture content of samples) of the standards. We consider them to be under-dimensioned because the moisture content of materials or products at the installation sites (adapted to the local atmospheric conditions) is usually higher as well as its fire resistance. This finding impacts mostly the economic efficiency (reducing costs) of buildings in the construction phase, when choosing the structural elements with required fire resistance, which could be according here presented findings lower.

## 5. Conclusions

This study dealt finding the relationship between the wood moisture content and parameters of charred layer, which is a product of pyrolysis. For this purpose, ANSYS software was applied. The transient thermal analysis has been the basis of all simulations. The following facts are the most significant findings of the research provided:

- The experimentally established thermal properties of wood entering the simulations in ANSYS represents a good basis in the process of a FE model development describing the European larch wood degradation rate in dependence of wood moisture content with the high correlation of numerical and experimental results (accuracy 93.4%).
- The formation of charred and degraded wood layers strongly depends on the moisture content in wood. The higher the moisture content, the smaller the volumes of these layers. Their volumes, in the range of wood MC from 10 to 30%, decrease exponentially with increasing moisture content.

## Data Availability

Not applicable.
